# Interparental Conflict and Early Adulthood Depression: Maternal Care and Psychological Needs Satisfaction as Mediators

**DOI:** 10.3390/ijerph19031402

**Published:** 2022-01-27

**Authors:** Shuangju Zhen, Jinjin Liu, Boyu Qiu, Lianying Fu, Jianping Hu, Binyuan Su

**Affiliations:** 1 Center for Studies of Psychological Application, School of Psychology, South China Normal University, Guangzhou 510631, China; jinjinliu@m.scnu.edu.cn (J.L.); boyuqiu@m.scnu.edu.cn (B.Q.); fulianying@m.scnu.edu.cn (L.F.); subinyuan@m.scnu.edu.cn (B.S.); 2 Key Laboratory of Brain, Cognition and Education Sciences, Ministry of Education, South China Normal University, Guangzhou 510631, China; 3 Guangdong Key Laboratory of Mental Health and Cognitive Science, South China Normal University, Guangzhou 510631, China; 4 Laboratory for Behavioral and Regional Finance, Guangdong University of Finance, Guangzhou 510631, China; jianpinghu@gduf.edu.cn

**Keywords:** interparental conflict, depression, antipathy, neglect, psychological needs satisfaction

## Abstract

Previous research has identified exposure to interparental conflict (IPC) in childhood as a risk factor for young adults’ depression. However, there is still a lack of understanding of the underlying mediating mechanisms of this association. Driven by the spillover hypothesis, the present study investigated whether maternal antipathy and neglect, and in turn unmet psychological needs, mediated the relation between IPC and early adulthood depression in a sample of 347 undergraduate students (M = 23.27 years; SD = 0.86; 57.05% women) in China. The participants completed self-report measures of IPC, maternal care, satisfaction of basic psychological needs, and depression. Structural equation modeling revealed that: (a) IPC was positively associated with early adulthood depression; (b) this association was sequentially mediated by inadequate maternal care (i.e., antipathy and neglect) and by unsatisfied psychological needs. These findings suggest that efforts to prevent depression should focus on reducing not only IPC, but also inadequate maternal care and unmet psychological needs.

## 1. Introduction

In recent years, Chinese college students’ depression has increasingly aroused social concern. According to recent data, the prevalence of Chinese college students’ depression was from 18.5% to 23.8% [1,2]. These statistics suggest that depression among college students cannot be ignored. Studies that focus on the underlying factors that influence depression are critical and have practical implications for personal mental health and interventions in early adulthood. The current study examined interparental conflict, and in turn, disruptions in parenting, in relation to depression in early adulthood.

### 1.1. Interparental Conflict and Depression

Interparental conflict (IPC) refers to the situation in which the parents cannot reach an agreement or the conflict between them becomes acute [3]. Although the prevalence and degree of children’s exposure to IPC was scarcely reported, statistics on the prevalence of children’s exposure to parental divorce and marital distress provided certain hints that the problem of IPC should be highlighted [4]. Between 2002 and 2019 in China, the divorce rate rose from 2.7 to 3.4 per 1000 people [5]. Due to incompatibility between partners, 77.5 percent of couples filed for divorce, whilst domestic violence accounted for 14.9 percent [6]. Unresolved and intense conflict may be upsetting to the child who witnesses or is involved in the situation [7]. Several studies have shown that exposure to IPC in childhood can predict later depression. For example, Westrupp et al. found that retrospective accounts of IPC during childhood were associated with children internalizing and externalizing problems in adolescence, reported that even a one-time report within the first six years of life can have negative effects on children’s mental health in adolescence [8].

A number of studies demonstrated that individuals who frequently witness marital relationship distress during early childhood are significantly more likely to suffer from depression and anxiety in adolescence [4,9,10]. Recent studies with similar results in China also showed that IPC was correlated with current depression in adolescents in junior high school [11,12] and in migrant children [13].

Thus, it appears that IPC does play a significant role in predicting later depression. Many studies have explored the underlying mechanism between IPC and depression. Keeports and Pittman found that young adults who reported more IPC also reported more symptoms of depression, with appraisals of threat and self-blame as parallel mediators [14]. Kim and Chung suggested that the student–teacher relationship mediated the association between IPC and depression [15]. Moreover, several studies specifically focused on the effect of family environment factors. For instance, Braithwaite et al. demonstrated that the parent–child relationship significantly mediated the relationship between IPC and internalizing problems such as depression [16]. Moreover, the mediating role of parent–child communication between IPC and depression was also investigated [17].

Based on the spillover hypothesis [18], interparental conflict and problematic parenting are causally linked. It illustrated that negativity in the parental subsystem spills over to the parent–child subsystem [19]. IPC may have a negative impact on children in part because it can leave parents emotionally drained [20] and less sensitive to their children’s needs [21].

A lack of parental care might prevent children’s psychological needs’ satisfaction and potentially lead to children’s psychological distress [22,23,24]. Given that Chinese research revealed a more significant role of mother’s parenting compared to that of father’s parenting on children’s development [12,25], the present study examined the roles of early maternal care and psychological needs satisfaction between IPC and young adult depression.

### 1.2. The Mediating Role of Inadequate Early Maternal Care: Maternal Antipathy and Neglect

Problems in parenting may partly explain the association between IPC in childhood and depression in young adulthood. Whiteside-Mansell et al. maintained that IPC may worsen the parents’ psychological status and stress levels, resulting in increased problematic parenting behaviors [26]. Smith et al. classified inappropriate early care for children into four dimensions: antipathy, neglect, sexual abuse, and physical abuse [27]. Among these, antipathy (gamma = 0.76) and neglect (0.73) [28] are highly associated with parents’ psychological attack, which is viewed as a major part of problematic parenting and discipline [29]. However, there are few studies on the relationship between unmet maternal care and depression. Indirect evidence comes from research showing that inadequate maternal care may fail to create the conditions needed for children to develop mental health [23].

In the current study, we assessed mothers’ parenting rather than fathers’ parenting since the mother–child relationship plays a more significant mediating role compared to the father–child relationship when IPC influences adolescents’ depression, especially in families with an only child [12]. Therefore, we focused on maternal antipathy and neglect to explore how inadequate early maternal care mediates the relationship between IPC and depression in adulthood.

### 1.3. The Mediating Role of Psychological Needs Satisfaction

Previous research revealed a negative relationship between IPC and psychological needs satisfaction [20]. According to the self-determination theory [30], one of the human fundamental needs is to experience feelings of security. Insufficient emotional support has been found to be a risk factor for children’s emotional insecurity [21] and negatively influence their emotional reactivity to stressors [31,32]. The theory of emotional security [33] may explain how IPC influences children’s psychological needs satisfaction. Emotional security reflects a feeling of confidence and safety in the family [33]. Children growing up in the context of conflict may develop feelings of insecurity that impairs their psychological needs satisfaction [33,34,35], and in response, these feelings may contribute to emotional and behavioral problems [36].

Furthermore, psychological needs satisfaction is considered essential for individuals to achieve psychological growth, self-identification, and well-being [30]. Several studies have shown that psychological needs satisfaction is associated with lower negative mood and lower depression among college students. In the U.S., college students whose basic psychological needs were met also showed more positive mood, more psychological vitality (feeling physically and mentally vigorous and alert), less negative mood, and fewer health complaints such as difficulty in breathing and soreness [37]. In China, college students’ needs satisfaction was shown to be associated with less depression [38], and a lack of needs satisfaction was shown to mediate the association between sexual assault victimization and depression [39]. Similar results were obtained in a study of Chinese college students [40,41]. This evidence supports the second part of the mediated pathway between IPC and young adults’ depression, namely the link between unmet psychological needs and depression. To explore the mediating role of basic psychological needs in the relationship between IPC and depression, the present study sought to provide a more comprehensive view of the correlations among these factors.

### 1.4. Inadequate Maternal Care and Unmet Basic Psychological Needs as Sequential Mediators

High levels of IPC between parents may lead to increased maternal antipathy and neglect through a process of spillover. These problems in parenting may prevent the satisfaction of individuals’ basic psychological needs and ultimately lead to depressive symptoms. Individuals who do not receive adequate maternal care and lack psychological needs satisfaction may behave passively and they may engage in counterproductive behaviors that ultimately hinder growth, internalization, or well-being [23,24]. Recent research showed that the relationship between cyber victimization and adolescents’ well-being was mediated by parental care and moderated by basic psychological needs satisfaction [42], indicating that parental care and psychological needs satisfaction are important in understanding the effects of other risk factors such as cyber victimization.

We propose that maternal antipathy and neglect are the immediate consequence of IPC, and unmet psychological needs satisfaction is a secondary effect. That is, the association between IPC and depression is mediated first by antipathy and neglect, and then by unmet psychological needs satisfaction. The results of tests of sequential mediation may inform strategies for preventing and intervening in adult depression.

### 1.5. The Current Study

**Figure 1:**
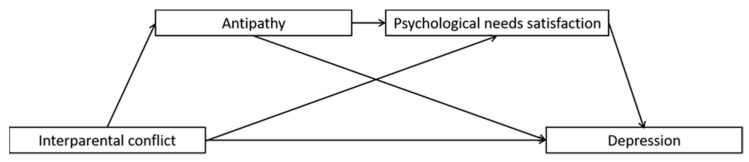
Full proposed model for the mediating roles of maternal antipathy and psychological needs satisfaction in the association between interparental conflict and depression.

**Figure 2:**
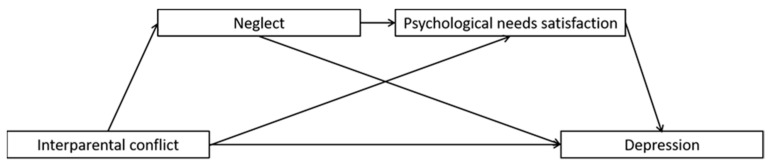
Full proposed model for the mediating roles of maternal neglect and psychological needs satisfaction in the association between interparental conflict and depression.

Children’s exposure to interpersonal conflict has been shown to be correlated with depression in young adulthood, but the reasons for this association are not clear. The present study aimed at investigating whether maternal antipathy and neglect and unmet psychological needs mediated the relation between IPC and early adulthood depression. College students completed questionnaire measures of all study variables. We used structural equation modeling to test two models, one involving maternal antipathy and the lack of basic psychological needs satisfaction as sequential mediators (Figure 1), and the other involving maternal neglect and the lack of basic psychological needs satisfaction as sequential mediators (Figure 2). We hypothesized that these sequential mediators would explain significant variance in the association between IPC and depression.

## 2. Materials and Methods

### 2.1. Participants

Questionnaires were collected from 347 undergraduate students in Guangdong Province, China, of which 345 were valid. The final sample consisted of 140 male students (42.95%) and 186 female students (57.05%), with a mean age of 23.27 years (SD = 0.86, range: 21–28 years). The study was approved by the Research Ethics Committee of the university with which the first author was affiliated.

### 2.2. Measures

#### 2.2.1. Interparental Conflict

IPC was measured by the Chinese version of the *Children’s Perception of the Interparental Conflict Scale* [43]. The Chinese version of the CPIC has been shown to be a reliable and valid measure of interparental conflict [44]. The scale includes three dimensions: frequency (e.g., “I often saw my mom and dad arguing or disagreeing”), intensity (e.g., “When mom and dad have an argument, they say mean things to each other”), and resolution (e.g., “When mom and dad disagree about something, they usually come up with a solution”). Respondents were asked to rate 17 items based on their experiences before age 12 on a four-point Likert scale from 1 (*False*) to 4 (*True*). Some of the item scores were reversed so that higher scores represented a higher level of interparental conflict. The mean of all item scores was calculated to create a composite interparental conflict score. In this study, the Cronbach’s alpha coefficient of the CPIC was 0.87.

#### 2.2.2. Early Maternal Care

Early maternal care was measured by 16 items from the *Childhood Experience of Care and Abuse Questionnaire* [27]. The Chinese version of the CECA.Q was translated by Li et al. [45]. Smith et al. reported that the dimensions of childhood antipathy (8 items referring to the amount of criticism, dislike or coldness shown by the mother towards the child) and neglect (8 items referring to neglect of the child’s material, social, education and emotional needs by the mother) from the CECA.Q were associated with the risk of depression in adulthood [27]. Therefore, the items reflecting these two dimensions were administered. Respondents were asked to indicate their experiences of maternal antipathy (e.g., “She was very critical of me”) and neglect (e.g., “She was not concerned about my whereabouts”) before age 12 on a five-point Likert scale from 1 (*Not at all*) to 5 (*Absolutely)*, with higher mean scores indicating poorer early maternal care. The Cronbach’s alpha coefficient of the total scale in this study was 0.87, including 0.82 for the antipathy subscale and 0.81 for the neglect subscale.

#### 2.2.3. Psychological Needs Satisfaction

The Chinese version [46] of the *Basic Psychological Needs Scale* [47] was used in the current study. The scale is based on Deci and Ryan’s [30] self-determination theory and measures the extent to which basic psychological needs are met in real-life situations. The scale includes 12 items, with 4 items for each of the three subdimensions: autonomy (e.g., “I feel like I am free to decide for myself how to live my life”), competence (e.g., “Often‚ I do not feel very competent”), and relatedness (e.g., “I get along with people I come into contact with”). Each item was rated on a five-point Likert scale from 1 (*Not at all*) to 5 (*Very much*). The mean score across all dimensions was calculated after reverse scoring some items, and higher scores indicated a higher level of psychological needs satisfaction. In the present study, the Cronbach’s alpha coefficient of the BPNS was 0.79.

#### 2.2.4. Depression

The Chinese version [48] of the *Beck Depression Inventory* [49] was used to access self-rating depression. The 21 items reflect a variety of symptoms and attitudes that are commonly found among clinically depressed individuals (e.g., “I am so sad or unhappy that I can’t stand it,” “I feel that the future is hopeless and that things cannot improve”). Respondents were asked to rate each item from zero to three, corresponding to the severity of the symptoms. The aggregate score ranges from 0 to 63, with higher scores indicating more symptoms of depression. The Cronbach’s alpha coefficient of the BDI was 0.91 in this study.

### 2.3. Procedure

The recruitment process began with the distribution of a brochure describing the project and an invitation to college students to participate. Written informed consent was obtained from each student. On the day of the assessment, trained researchers distributed self-report questionnaires to students in their classrooms. The confidentiality of the study was emphasized at the beginning of the collection process. Participation required completing the questionnaires in person. The participants were told that they could withdraw their questionnaire at any time during the data collection period, without penalty.

### 2.4. Statistical Approach

The mediating effects were estimated using structural equation modeling in Mplus 7.0 [50]. The full-information maximum likelihood estimation procedure was applied to accommodate missing data. Bootstrap procedures were used to test and validate the statistical significance of the paths. The bootstrap procedure computes an estimation of the indirect effect with a 95% Confidence Interval (CI). The indirect effect is considered significant when zero is excluded from the confidence interval.

## 3. Results

### 3.1. Descriptive Statistics

Means, standard deviations, and correlations across all variables are presented in Table 1. IPC, antipathy and neglect were positively correlated with depression (*r*_1_ = 0.31, *p* < 0.001; *r*_2_ = 0.38, *p* < 0.001; *r*_3_ = 0.12, *p* < 0.05), indicating that they might be risk factors for depression. Psychological needs satisfaction was negatively correlated with depression (*r* = −0.53, *p* < 0.001), suggesting that students whose basic psychological needs are fulfilled will have milder depressive symptoms. All variables were significantly correlated with each other.

### 3.2. The Mediating Roles of Antipathy and Psychological Needs Satisfaction in the Association between IPC and Depression

**Figure 3:**
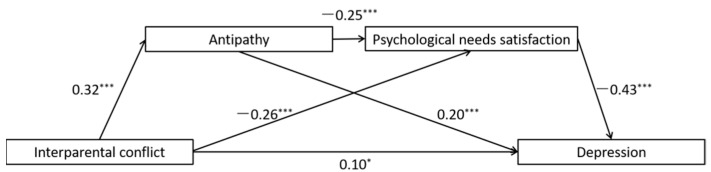
Results of the full proposed model for the mediating roles of antipathy and psychological needs satisfaction in the association between interparental conflict and depression. Note: significant standardized paths are displayed by the solid lines. *** *p* < 0.001, * *p* < 0.05.

**Figure 4:**
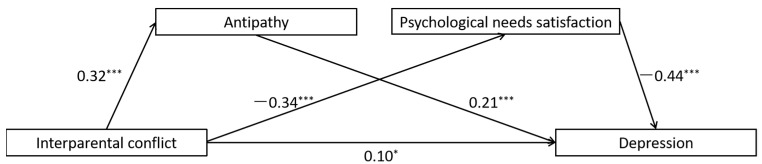
Results of the alternative model for the mediating roles of antipathy and psychological needs satisfaction in the association between interparental conflict and depression. Note: significant standardized paths are displayed by the solid lines. *** *p* < 0.001, * *p* < 0.05.

The full proposed model was constructed, including: (a) the direct path: IPC → depression, (b) the indirect path for antipathy: IPC → antipathy → depression, (c) the indirect path for psychological needs satisfaction: IPC → psychological needs satisfaction → depression, (d) the direct path for mediators: antipathy → psychological needs satisfaction. All the paths in the full proposed model were statistically significant (See Figure 3 for standardized path coefficients). To ascertain the direct path from antipathy to psychological needs satisfaction, we deleted the path between them (antipathy → psychological needs satisfaction), and the chi-square difference test showed that the model fit became significantly worse after deleting the path, *χ*^2^(*df* = 1) = 22.83, *p* < 0.001 (See Figure 4 for standardized path coefficients).

In accordance with the hypotheses, (a) higher IPC significantly predicted more maternal antipathy (*b* = 0.32, *p* < 0.001), which in turn predicted more severe depression symptoms (*b* = 0.20, *p* < 0.001); (b) higher interparental conflict significantly predicted psychological needs satisfaction (*b* = −0.26, *p* < 0.001), and psychological needs satisfaction in turn predicted more severe symptoms of depression (*b* = −0.43, *p* < 0.001); and (c) more maternal antipathy significantly predicted psychological needs satisfaction (*b* = −0.25, *p* < 0.001).

The indirect effects are reported in Table 2. Bootstrapping analyses suggested that antipathy alone, psychological needs satisfaction alone, and the combination of antipathy and psychological needs satisfaction were all significant mediators in the association between IPC and depression (indirect effects = 0.064, 0.110, 0.034, respectively; *p* < 0.05).

### 3.3. The Mediating Role of Neglect and Psychological Needs Satisfaction on the Association between IPC and Depression

**Figure 5:**
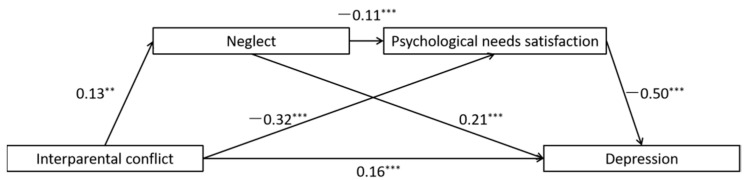
Results of the full proposed model for the mediating roles of neglect and psychological needs satisfaction in the association between interparental conflict and depression. Note: significant standardized paths are displayed by the solid lines. *** *p* < 0.001, ** *p* < 0.01.

**Figure 6:**
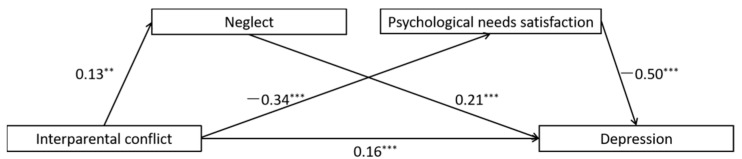
Results of the alternative model for the mediating roles of neglect and psychological needs satisfaction in the association between interparental conflict and depression. Note: significant standardized paths are displayed by the solid lines. *** *p* < 0.001, ** *p* < 0.01.

The full proposed model was constructed, including: (a) the direct path: IPC → depression, (b) the indirect path for neglect: IPC → neglect → depression, (c) the indirect path for psychological needs satisfaction: IPC → psychological needs satisfaction → depression, (d) the direct path for mediators: neglect → psychological needs satisfaction. All the paths in the full proposed model were statistically significant (See Figure 5 for standardized path coefficients). To ascertain the direct path from neglect to psychological needs satisfaction, we deleted the path between them (neglect → psychological needs satisfaction), and the chi-square difference test showed that the model fit became significantly worse after deleting the path, *χ*^2^(*df* = 1) = 4.48, *p* = 0.03 (See Figure 6 for standardized path coefficients).

In accordance with the hypotheses, (a) higher IPC significantly predicted more maternal neglect (*b* = 0.13, *p* = 0.004), which in turn predicted more severe symptoms of depression (*b* = 0.21, *p* < 0.001); (b) higher interparental conflict significantly predicted less psychological needs satisfaction (*b* = −0.32, *p* < 0.001), and in turn predicted more severe symptoms of depression (*b* = −0.50, *p* < 0.001); and (c) more maternal neglect significantly predicted less psychological needs satisfaction (*b* = −0.11, *p* < 0.001).

Finally, the indirect effects are reported in Table 3. Bootstrapping analyses indicated that neglect alone, psychological needs satisfaction, and the sequential effects of neglect and lack of basic psychological needs satisfaction were sequential mediators of the association between interparental conflict and depression (indirect effects = 0.028, 0.163, 0.07, respectively; *p* < 0.05).

## 4. Discussion

The current study investigated whether and how perceived IPC in childhood and adolescence is associated with depressive symptoms in early adulthood. The research was conducted in a sample of university students in China. Tests of multiple mediator models showed that a higher level of IPC was related to more maternal antipathy and neglect and less psychological needs satisfaction, and in turn, more depressive symptoms in early adulthood. These findings expand existing empirical knowledge about the potential influence of IPC on depressive symptoms and provide valuable insights for intervention.

In accordance with existing research [17,51] and a systematic review [52], the results suggest a positive relationship between IPC and depressive symptoms. Regarding the essential roles that parents play in the family system during the formative periods of their children’s lives [17], the enduring negative effects of IPC are evident in the domain of children’s mental health [53,54], for the reason that parents may be less sensitive and emotionally responsive to their children’s needs [21]. As a result, children who are confronted with IPC may be more likely to endure depressive symptoms. Furthermore, the current results extend the scope of extant research by examining these processes during the developmental period of early adulthood.

With regard to the underlying mechanism, the results demonstrated that a higher level of IPC was significantly related to more maternal antipathy and neglect, which in turn predicted more symptoms of depression. In accordance with the spillover hypothesis, IPC has been shown to be associated with parenting disturbances, such as harshness, inconsistency, and psychological intrusiveness [55], hostility and sensitivity [56], and parental distress [57]. The current results suggested that IPC was also associated with maternal antipathy and neglect. Although the associations were significant in both cases, the coefficients involving antipathy were larger than those involving neglect. It seems that maternal antipathy may play a more important role than neglect does. This pattern is consistent with several other studies suggesting the relative importance of antipathy in predicting a range of outcomes. Maternal antipathy, relative to neglect, has been shown to be a stronger predictor of externalizing and internalizing problems, such as youths’ nonsuicidal self-injury [58], as well as lower self-report authenticity and higher cross-context trait variability [59]. Furthermore, Kernis and Goldman have demonstrated that children who perceived antipathy are more likely to suppress their true selves, ignore or conceal their expressions in favor of others in order to seek approval from parents, which may lead to a higher risk of internalizing problems [60]. Therefore, further prevention and intervention for depression should address IPC and attendant maternal care, especially maternal antipathy.

As predicted, IPC was positively related to psychological needs satisfaction, which in turn was negatively related to depressive symptoms. Furthermore, more maternal antipathy and neglect were related to lower psychological needs satisfaction. Notably, IPC predicts a lack of parental care, and obstructive parenting behaviors will prevent needs satisfaction [23,24], potentially leading to psychological distress. In contrast, parents who provide supportive parental care (e.g., autonomy support, structure, and warmth) meet children’s basic psychological needs, promoting needs satisfaction and adjustment [22]. The findings in the current study suggest that the perception of IPC may exert an indirect impact through a lack of psychological needs satisfaction, and these unmet needs may increase the risk of depression.

The present study has several limitations. First, the findings in this study were based on a cross-sectional design. Although the hypotheses were based on theory and solid empirical evidence, causality cannot be inferred. Future research should use longitudinal designs to seek more evidence of causality. Second, data collection was based on self-report by the participants in a retrospective way. Although previous research showed that college students’ perception of early negative experiences was rather accurate and stable over time [61], future researchers may also collect data from parents longitudinally. Third, the depression scores of our participants were low. It might be helpful for future research to examine the relationship between IPC and depression in a clinical sample. Fourth, our study had not examined father’s parenting and how it works on children’s depression. Further study may collect data from both parents and explore whether there is a difference between mother’s and father’s parenting on children’s depression.

## 5. Conclusions

The current study highlights the role of several aspects of family life as risk factors for the development of depression in young adults. The results showed that perceived IPC earlier in life related to depression in early adulthood through the mediating roles of maternal antipathy, maternal neglect, and unmet psychological needs. In addition, there was a sequential mediation effect in which IPC was associated with both antipathy and neglect, which were in turn related to a lack of psychological needs satisfaction and more developed depressive symptoms in young adulthood. By clarifying this developmental process, the study provides evidence that will be helpful in designing effective prevention and intervention programs in the future.

## Figures and Tables

**Table 1:** Descriptive statistics and correlations for all variables.

Variable	M	SD	1	2	3	4	5
1. IPC	2.19	0.54	1				
2. EMC: Antipathy	1.85	0.67	0.32 ***	1			
3. EMC: Neglect	2.07	0.92	0.13 *	0.27 ***	1		
4. PNS	3.47	0.55	−0.34 ***	−0.33 ***	−0.15 **	1	
5. Depression	8.82	7.31	0.31 ***	0.38 ***	0.12 *	−0.53 ***	1

Note: *** *p* < 0.001, ** *p* < 0.01, * *p* < 0.05. IPC: Interparental conflict, retrospective report of the intensity of conflict between parents; EMC: Early maternal care, retrospective report of maternal care before age 12; PNS: Psychological needs satisfaction, retrospective report of the account of current need satisfaction; Antipathy: Hostility or other negative emotions from the mother; Neglect: The mother’s indifference on material care or other needs for healthy development.

**Table 2:** Indirect effects for the mediating roles of antipathy and psychological needs satisfaction in the association between IPC and depression.

Path	*b*	95% CI
IPC → antipathy → depression	0.064	0.033, 0.095
IPC → psychological needs satisfaction → depression	0.110	0.068, 0.153
IPC → antipathy → psychological needs satisfaction → depression	0.034	0.017, 0.051

Note: IPC: Interparental conflict, retrospective report of the intensity of conflict between parents.

**Table 3:** Indirect effects for the mediating roles of neglect and psychological needs satisfaction in the association between IPC and depression.

Path	*b*	95% CI
IPC → neglect e depression	0.028	0.010, 0.046
IPC →psychological needs satisfaction → depression	0.163	0.115, 0.211
IPC → neglect → psychological needs satisfaction → depression	0.007	0.001, 0.014

Note: IPC: Interparental conflict, retrospective report of the intensity of conflict between parents.

## Data Availability

The data presented in this study are available on request from the corresponding author.
